# Performance of the multi-target hepatocellular carcinoma blood test: Design and rationale of the ALTUS study

**DOI:** 10.1097/HC9.0000000000000800

**Published:** 2025-10-07

**Authors:** Binu V. John, Mark Camardo, Kyle Porter, Paul Z. Elias, Elle Kielar-Grevstad, Seema P. Rego, Neehar D. Parikh, Lewis R. Roberts, Alvin C. Silva, Amit G. Singal, Ju Dong Yang, Paul J. Limburg

**Affiliations:** 1 Division of Hepatology, Miami VA Medical Center, Miami, Florida, USA; 2 Department of Medicine, University of Miami Miller School of Medicine, Miami, Florida, USA; 3 Exact Sciences Corporation, Madison, Wisconsin, USA; 4 Division of Gastroenterology and Hepatology, Department of Internal Medicine, University of Michigan, Ann Arbor, Michigan, USA; 5 Division of Gastroenterology and Hepatology, Department of Internal Medicine, Mayo Clinic, Rochester, Minnesota, USA; 6 Department of Radiology, Mayo Clinic, Scottsdale, Arizona, USA; 7 Division of Digestive and Liver Diseases, Department of Internal Medicine, University of Texas Southwestern Medical Center, Dallas, Texas, USA; 8 Samuel Oschin Comprehensive Cancer Institute, Cedars-Sinai Medical Center, Los Angeles, California, USA; 9 Karsh Division of Gastroenterology and Hepatology, Cedars-Sinai Medical Center, Los Angeles, California, USA; 10 Comprehensive Transplant Center, Cedars-Sinai Medical Center, Los Angeles, California, USA

**Keywords:** alpha-fetoprotein, hepatocellular carcinoma blood test, hepatocellular carcinoma screening, hepatocellular carcinoma surveillance, liver ultrasound

## Abstract

**Background:**

Hepatocellular carcinoma (HCC) is a leading cause of cancer-related death in the United States and worldwide. HCC screening among patients at elevated risk is associated with improved early detection and overall survival, but current ultrasound-based screening strategies are challenged by low adherence and suboptimal sensitivity. Blood-based screening tests have the potential to enhance adherence and improve early-stage HCC detection if they can demonstrate favorable performance compared with ultrasound-based screening. Here we present the design and rationale for ALTUS (*A* *l*ternative *t*o *U*ltra*s*ound), a prospective, longitudinal, multicenter study in the United States to investigate the performance of the multi-target HCC blood test (mt-HBT) for the detection of HCC in a screening population.

**Methods:**

Adults with liver cirrhosis or chronic hepatitis B infection will be enrolled to undergo standard-of-care screening imaging and concurrent blood collection for the mt-HBT. All participants will undergo contrast-enhanced CT or MRI imaging with central radiology LI-RADS assessment as the reference method to determine HCC status. Participants without an identified malignancy will undergo a second screening visit and blood collection, and longitudinal clinical and imaging data will be collected up to 18 months from enrollment. The primary study objectives are to demonstrate that the mt-HBT is non-inferior to ultrasound for early-stage HCC sensitivity and to assess mt-HBT HCC specificity. The secondary objective is to assess mt-HBT overall sensitivity.

**Results:**

ALTUS is in progress with readout of the primary analysis expected in 2025.

**Conclusions:**

This prospective head-to-head comparison of the mt-HBT versus ultrasound will provide novel data regarding the performance and utility of the mt-HBT for HCC screening.

## INTRODUCTION

Liver cancer is the sixth leading cause of cancer-related death in the United States, with ~30,090 deaths projected for 2025.^1^ Liver cancer is also the fastest growing cause of cancer-related deaths and is projected to surpass breast and colorectal cancer by 2040.^2^ Hepatocellular carcinoma (HCC) accounts for ~90% of liver cancers and frequently arises in the background of chronic liver diseases such as hepatitis C, hepatitis B, alcohol-associated liver disease (ALD), and metabolic dysfunction–associated steatotic liver disease (MASLD).^3^^,^^4^ Cirrhosis is the predominant risk factor for HCC development and is present in 80%–90% of patients with HCC.^3^^,^^4^ Current clinical practice guidelines recommend biannual HCC screening using abdominal ultrasound, with or without serum alpha-fetoprotein (AFP) measurement, in at-risk patients with cirrhosis and select patients with chronic HBV infection without cirrhosis.^4^^–^^8^ Although HCC screening is associated with improved early detection and a greater likelihood of curative treatment, the sensitivity for early-stage HCC has been estimated at only 47% for ultrasound alone, and 63% for ultrasound plus AFP.^9^^,^^10^ Ultrasound sensitivity for early-stage HCC in studies in the United States has been estimated at only 36%.^10^ Ultrasound performance may be diminished by factors such as variable operator expertise and poor visualization in patients with obesity, liver nodularity, or liver steatosis.^11^^–^^15^ Ultrasound sensitivity may also be limited in small lesions, as well as for infiltrative lesions without a clear boundary. Visualization limitations are likely to become increasingly common with the increasing prevalence of MASLD.^16^^,^^17^ In addition, screening utilization in patients at risk of HCC in the United States is low and estimated to be <24% due to a variety of patient-related and provider-related barriers, including access to imaging.^18^^–^^21^ Low adherence to recommended ultrasound-based screening, coupled with variable and sub-optimal early-stage HCC sensitivity, limits the effectiveness of current screening programs and highlights the need for improved strategies.^22^

A promising approach to early HCC detection is the analysis of blood-based biomarkers, sometimes referred to as “liquid biopsy.”^22^ In an international multicenter case–control study, Chalasani et al^23^ previously described the performance characteristics of the multi-target HCC blood test (mt-HBT), which incorporates measurement of methylated DNA markers, AFP, and patient sex into an algorithm to qualitatively evaluate the likelihood of HCC. The mt-HBT generates a binary result of “Negative” or “High,” where High indicates an elevated likelihood of HCC. In a case–control validation cohort, the mt-HBT demonstrated 82% sensitivity for early-stage HCC [Barcelona Clinic Liver Cancer (BCLC) 0 and A], 88% overall sensitivity, and 87% specificity. However, prospective validation is required to assess test performance in the intended use population.^24^ Further, a direct assessment against ultrasound-based screening will enable a better understanding of the comparative performance and potential value of the mt-HBT. We present here the design and rationale of ALTUS (*Al*ternative *t*o *U*ltra*s*ound), a prospective study to establish the clinical performance of the mt-HBT for the detection of HCC in patients at increased risk.

## METHODS

### Study design and conduct

ALTUS is a prospective, longitudinal, multicenter study in the United States to establish the clinical performance of the mt-HBT for the detection of HCC in patients with cirrhosis or chronic HBV infection. To assess whether the mt-HBT provides a valuable alternative to ultrasound-based HCC screening, ALTUS includes a head-to-head non-inferiority comparison between mt-HBT and ultrasound for early-stage HCC sensitivity. HCC status will be determined by multiphase contrast-enhanced CT or MRI with central radiology assessment, or by pathology when available.

ALTUS will be conducted in accordance with the Declaration of Helsinki, ICH GCP E6 (R2), ISO 20916:2019, applicable national, state, and/or local laws and regulations, and conditions of approval imposed by the reviewing Institutional Review Board (IRB). Written consent will be obtained from all participants. ALTUS is registered with clinicaltrials.gov (NCT05064553).

### Study definitions

For participants undergoing ultrasound screening, imaging will be assessed locally using v2017 Ultrasound LI-RADS.^25^ Ultrasound results will be classified for the study as Ultrasound Positive for US-3 LI-RADS assessments, or Ultrasound Negative for US-1 and US-2 LI-RADS assessments. The HCC status of all participants will then be determined by multiphase contrast-enhanced CT/MRI imaging with central radiology assessment using v2018 CT/MRI Diagnostic LI-RADS,^26^ or by definitive pathology when available. HCC status will be classified for the study as confirmed HCC, confirmed non-HCC, or indeterminate observation. Early-stage HCC will be defined by the Milan criteria.^27^ Although BCLC staging is widely used and will also be reported in ALTUS, it includes Eastern Cooperative Oncology Group (ECOG) performance status and Child–Pugh status, which are independent of tumor burden. Improvements in cancer screening efficacy using blood-based biomarkers can impact tumor burden at the time of diagnosis, but not liver function or patient performance status. Milan criteria will likely yield more objective data for the primary analysis, as it is determined by tumor burden alone and can be fully assessed using cross-sectional imaging. The category of confirmed HCC will be restricted to pathology-confirmed HCC or central radiology classifications of LR-5 or LR-TIV with assessment of definite or probable HCC. The algorithm determining the mt-HBT test result of “Negative” or “High” was developed in previous work and locked before the evaluation of ALTUS results.^23^ A full list of study definitions is provided in Table 1.

**TABLE 1:** ALTUS study definitions

Study term	Definition
Ultrasound positive^a^	• US-3 (Positive)
Ultrasound negative^a^	• US-1 (Negative) • US-2 (Subthreshold)
mt-HBT high	• mt-HBT result indicating an elevated likelihood of HCC
mt-HBT negative	• mt-HBT result indicating a lower likelihood of HCC
Confirmed HCC^b^ ^c^	• Pathology-confirmed HCC or cHCC-CCA, regardless of LI-RADS category • LR-5 • LR-TIV with definite or probable HCC^d^
Confirmed non-HCC^b^ ^c^	• Pathology-confirmed malignancies other than HCC or cHCC-CCA • Pathology-confirmed benign observations • No observation/LR-Negative • LR-1 • LR-2
Indeterminate observation^b^	• LR-NC (not categorizable) • LR-3 • LR-4 • LR-M • LR-TIV without definite or probable HCC^4^
Early-stage HCC (Milan criteria^e^)	• Single lesion ≤5 cm, or • Up to 3 lesions, each ≤3 cm • No macrovascular invasion or extrahepatic metastases

^a^ Ultrasound classifications will be based on local radiology review using v2017 Ultrasound LI-RADS.

^b^ CT/MRI classifications will be based on central radiology review using v2018 CT/MRI Diagnostic LI-RADS.

^c^ Histopathology interpretation will be conducted according to standard practice at local sites.

^d^ Assessment of HCC probability for LR-TIV observations is based on central radiology interpretation of LI-RADS features.

^e^ Although some interpretations of the Milan criteria exclude solitary lesions <2 cm and multifocal lesions each < 1 cm, such lesions will be considered within the Milan criteria for this analysis.^27^^–^^29^

Abbreviations: cHCC-CCA, combined hepatocellular-cholangiocarcinoma; mt-HBT, multi-target hepatocellular carcinoma blood test; LI-RADS, liver imaging reporting and data system; LR, LI-RADS, used as a descriptor with a LI-RADS category; LR-M, liver imaging reporting and data system - malignancy; LR-TIV, liver imaging reporting and data system-tumor in vein; US, ultrasound.

### Study objectives

The value of an HCC screening test is primarily in the ability to detect cancers at an early stage when treatment with curative intent (eg, resection, ablation, or transplant) is possible.^9^ As such, the primary objective of ALTUS is to demonstrate in a head-to-head comparison that mt-HBT is non-inferior to ultrasound for early-stage HCC sensitivity within a 5% margin.

In addition to detecting early-stage disease, a viable screening test should reasonably limit the number of false positive results, which may lead to unnecessary diagnostic imaging and potential associated harms.^22^ A co-primary objective is therefore to assess mt-HBT HCC specificity.

Although early-stage detection is the main goal of screening, there is also potential benefit in detecting HCCs that have progressed beyond early-stage. For example, downstaging therapies may reduce tumor burden to allow transplant eligibility among patients with tumors initially outside Milan or other transplant criteria.^4^ The secondary objective of ALTUS is therefore to assess mt-HBT overall HCC sensitivity.

A number of additional prespecified analyses will be conducted, including but not limited to: calculation of mt-HBT predictive values and likelihood ratios, mt-HBT performance with stratification by BCLC staging and tumor size, mt-HBT performance with stratification by demographics and clinical characteristics, mt-HBT performance in participants with indeterminate or suspicious radiology observations (ie, LR-3, LR-4 observations), mt-HBT performance incorporating test results from multiple blood collections and longitudinal resolution of HCC clinical status over the follow-up period, mt-HBT performance comparisons with AFP and AFP plus ultrasound, and assessment of concordance and discordance between local and central radiology assessments. The ALTUS study objectives are summarized in Table 2.

**TABLE 2:** ALTUS study objectives

ALTUS study objectives	
Primary objectives	• To demonstrate that early-stage HCC sensitivity of mt-HBT is non-inferior to that of ultrasound within a 5% margin • To assess overall specificity of mt-HBT for HCC
Secondary objective	• To assess the overall sensitivity of mt-HBT for HCC
Other prespecified analyses	• mt-HBT predictive values, likelihood ratios, and AUROC • mt-HBT performance with stratification by BCLC staging and tumor size (<2 cm vs. 2–3 cm vs. 3–5 cm)^30^ • mt-HBT performance with stratification by demographics and clinical characteristics (eg, body mass index, liver disease etiology, Child–Pugh score) • mt-HBT performance in indeterminate radiology observations with longitudinal resolution of HCC status • mt-HBT performance incorporating results from multiple blood collections and longitudinal resolution of HCC clinical status over the follow-up period • mt-HBT performance comparison to AFP and AFP + ultrasound • Local and central radiology concordance and discordance

Abbreviations: AFP, alpha-fetoprotein; BCLC, Barcelona Clinic Liver Cancer; mt-HBT, multi-target hepatocellular carcinoma blood test.

### Study population

#### Study sites and sample size

ALTUS will enroll ~3000 participants from more than 50 sites across the United States, including academic and Veterans Affairs–based medical centers, community outpatient clinics, and independent research centers.

#### Inclusion criteria and exclusion criteria

Eligibility criteria include participants being 18 years of age or older and presenting for HCC screening due to cirrhosis or chronic HBV infection. Cirrhosis will be established based on biopsy, imaging evidence of cirrhosis with concomitant laboratory, imaging, or endoscopic evidence of portal hypertension, or by elevated liver stiffness measurement on MR or transient elastography.^31^

Exclusion criteria include known hepatic or non-hepatic cancer diagnosis within the past 5 years, Child–Pugh Class C cirrhosis at enrollment in a patient who is not a transplant candidate, prior finding of a liver nodule >1 cm by ultrasound or elevated AFP (>100 ng/mL) within 12 months of enrollment, pregnancy, major illness with high risk of mortality during the study period, sustained virologic response (SVR) for HCV for ≥10 years before enrollment,^32^^,^^33^ or an inability to undergo contrast-enhanced CT or MRI. Full inclusion and exclusion criteria are listed in Table 3.

**TABLE 3:** ALTUS inclusion and exclusion criteria

Inclusion criteria 1. 18 y of age or older 2. Understands the study procedures and is able to provide written informed consent to participate in the study, and has authorization for release of data, including personal health data and images, to the study Investigator, Sponsor, and regulatory authorities. 3. Presents for surveillance imaging due to increased risk for HCC, including: i. Diagnosis of cirrhosis based on at least one of the following: a. Histology from a liver biopsy b. Ultrasound, CT, or MRI showing a cirrhotic liver combined with portal hypertension [as evidenced by the presence of intra-abdominal varices, recanalized umbilical vein, ascites, splenomegaly, or thrombocytopenia (defined as platelet count <150,000)]. The imaging results must have been obtained within 5 y of study enrollment. c. Liver stiffness ≥4.71 kPa by MR elastography or ≥12.1 kPa by vibration-controlled transient elastography d. Presence of varices on endoscopy or imaging, and presence of a chronic liver disease. Endoscopy or imaging results must have been obtained within 5 y of study enrollment. ii. Chronic HBV infection (hepatitis B surface antigen present for >6 mo) without cirrhosis.	Exclusion criteria 1. Known cancer diagnosis (including active malignancy) within the past 5 y except for non-melanoma skin cancer. 2. Chemotherapy and/or radiation therapy within 5 y before study enrollment. 3. Known Child–Pugh class C liver function at the time of enrollment, except for those on the waiting list for transplant. 4. Solid liver nodule >1 cm by ultrasound or elevated AFP (>100 ng/mL) in 12 mo preceding the qualifying surveillance imaging visit without subsequent documentation of HCC negative or LI-RADS 1 by diagnostic CT/MRI. 5. Females known to be pregnant at the time of enrollment. 6. Illness that the investigator believes poses a significant risk of mortality during the study period, including but not limited to i. Congestive heart failure with ejection fraction <50% ii. Chronic lung disease requiring supplemental oxygen. iii. History of recent stroke. 7. Sustained virologic response (SVR) for HCV (undetectable HCV RNA 12–24 wk after completion of antiviral therapy) for >10 y before enrollment. 8. Not able to have IV contrast for CT or MRI due to i. Allergy to IV contrast and unwilling or unable to receive IV contrast after pre-medication. ii. Estimated glomerular filtration rate <35 mL/min and not on hemodialysis.

Abbreviations: CT/MRI, computed tomography/magnetic resonance imaging; HCV RNA, hepatitis C virus ribonucleic acid; IV, intravenous; MR, magnetic resonance.

#### Study population distribution by screening modality, cirrhosis status, and age

Although ultrasound is the imaging modality recommended in the National Comprehensive Cancer Network (NCCN) and the American Association for the Study of Liver Diseases (AASLD) guidelines for routine HCC screening in most circumstances,^5^^,^^6^ CT and MRI are sometimes used for screening in practice.^4^^,^^34^ A limited proportion (<10%) of participants undergoing CT/MRI screening will therefore be included in the study to help ensure a representative study population for estimating mt-HBT overall HCC sensitivity and mt-HBT HCC specificity. However, these participants will be excluded from the primary analysis comparing early-stage HCC sensitivity of the mt-HBT with ultrasound. The ultrasound screening arm will comprise ≥90% of participants and will be used to assess mt-HBT non-inferiority to ultrasound for early-stage HCC sensitivity. Participants with chronic HBV infection without cirrhosis will be limited to no more than 10% of the total study enrollment, as 80%–90% of HCC cases in the US occur in patients with cirrhosis.^3^^,^^4^ Considering that patient populations with cirrhosis may have a relatively high proportion of older individuals^35^^,^^36^ and HCC incidence generally increases with age,^36^^–^^38^ the study will aim for a prespecified age distribution with at least 80% of participants age 55 or older.

### Study procedures and workflow

#### Enrollment and screening Visit 1

**FIGURE 1:**
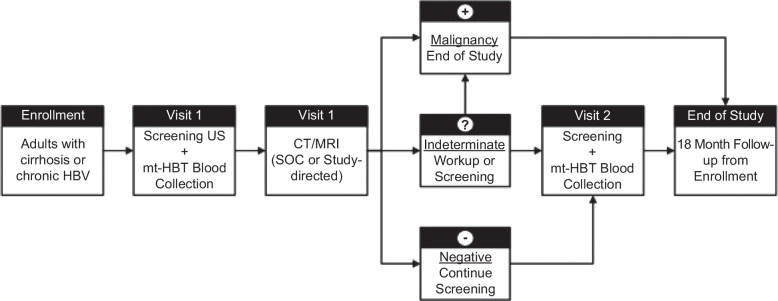
Clinical study workflow for participants receiving standard-of-care ultrasound (US) screening at Visit 1. Abbreviations: mt-HBT, multi-target hepatocellular carcinoma blood test; SOC, standard of care.

**FIGURE 2:**
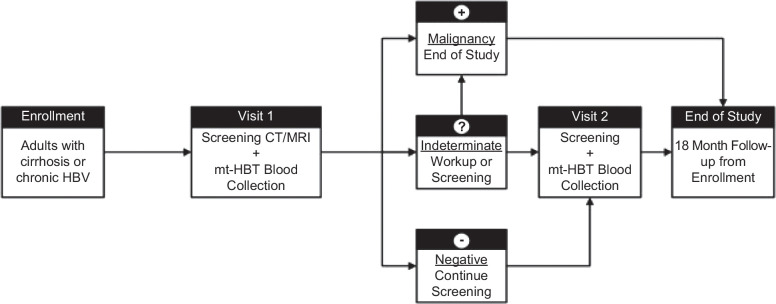
Clinical study workflow for participants receiving standard-of-care CT/MRI screening at Visit 1. Abbreviation: mt-HBT, multi-target hepatocellular carcinoma blood test.

Eligible participants will be enrolled to undergo standard-of-care screening imaging (ultrasound or CT/MRI) and concurrent blood collection for the mt-HBT at Visit 1. Participants receiving ultrasound screening (Figure 1) will undergo a subsequent contrast-enhanced multiphase CT/MRI (standard-of-care diagnostic imaging after an ultrasound-positive result or study-directed imaging after an ultrasound-negative result) for local patient management and to serve as the reference imaging method for the study. Blood collection and imaging procedures are to be completed within 30 days. For participants undergoing standard-of-care multiphase contrast-enhanced CT/MRI screening (Figure 2), the screening CT/MRI will serve as the study reference imaging. All CT/MRI imaging will undergo central radiology assessment (2 independent readers with independent adjudication of discrepancies by a third reader) to establish the reference HCC status for mt-HBT performance and ultrasound performance. However, local pathology obtained within 6 months of the initial screening visit will serve as the definitive reference result for HCC status if available. Central radiology reviewers will remain blinded to local CT/MRI and ultrasound results. Participants, providers, and central radiology reviewers will remain blinded to mt-HBT results, which will not be used in clinical management.

#### Screening Visit 2

Participants without a local malignancy diagnosis from Visit 1 are anticipated to return within 8 months of the first visit for a second standard-of-care screening visit with a second blood collection for the mt-HBT. Although Visit 2 will not require reference CT/MRI imaging for all participants, any CT/MRI imaging ordered by local treating physicians will undergo the same central radiology process as Visit 1, with all blinding rules in effect. Visit 2 will support exploratory analyses related to the resolution of indeterminate observations and the evaluation of mt-HBT performance using results from multiple blood collections.

#### Longitudinal follow-up

Clinical, lab, and imaging data, including standard-of-care screening imaging visits, will be collected for each participant up to 18 months from enrollment. Any CT/MRI imaging performed as part of clinical care during the longitudinal follow-up period will be collected and will undergo the same central radiology review process as in Visit 1 and Visit 2. For participants diagnosed with HCC, treatment and outcomes data will be collected during the follow-up period.

### Analysis

#### Sample size and statistical power

The sample size was driven by the target of 26 early-stage HCC cases required to have at least 80% power for the primary non-inferiority analysis. With ~3000 participants, the study is expected to yield at least 26 early-stage HCC cases and have >80% power for the primary non-inferiority analysis and >95% power for the primary specificity analysis at 2-sided 5% significance levels, assuming overall HCC incidence of 1.7% per year,^35^^,^^36^^,^^39^^,^^40^ early-stage HCC incidence of 1.1% per year,^9^^,^^41^ mt-HBT early-stage HCC sensitivity of 65%,^23^ and ultrasound early-stage HCC sensitivity of 35%,^10^ along with estimates for proportion enrolled in the ultrasound screening arm, rate of withdrawals and unusable samples, and concordance between ultrasound and mt-HBT.

#### Non-inferiority comparison of the mt-HBT to ultrasound for early-stage HCC sensitivity

All eligible and evaluable participants with a valid mt-HBT result, a confirmed early-stage HCC reference result, and a paired ultrasound result will be included in the primary non-inferiority analysis. This analysis will include only confirmed early-stage HCC cases among participants in the ultrasound screening arm and will be based on Visit 1 results. Ultrasound early-stage HCC sensitivity will be evaluated as the proportion of ultrasound-positive results among participants with confirmed HCC status meeting Milan criteria. mt-HBT early-stage HCC sensitivity will be evaluated as the proportion of mt-HBT High results among participants with confirmed HCC status meeting Milan criteria. Non-inferiority of the mt-HBT to ultrasound for early-stage HCC sensitivity will be tested using a 5% non-inferiority margin. The two-sided 95% Tango Score confidence interval^42^ for the difference between the mt-HBT and ultrasound early-stage HCC sensitivity will be calculated, taking the paired nature of the data into account. If the lower bound of the confidence interval is greater than −5%, non-inferiority to ultrasound will be concluded. If non-inferiority is demonstrated, superiority of the mt-HBT to ultrasound will be tested using the McNemar test.

#### Assessment of mt-HBT HCC specificity

All eligible and evaluable participants with a valid mt-HBT result and a confirmed non-HCC reference result will be included in the primary specificity analysis. This analysis will include all confirmed non-HCC cases in the ultrasound and CT/MRI screening arms based on Visit 1 results. mt-HBT HCC specificity will be evaluated as the proportion of mt-HBT negative results among participants with confirmed non-HCC status. A point estimate with a 2-sided Wilson score 95% CI will be calculated for mt-HBT HCC specificity, and the lower bound will be compared with a prespecified performance benchmark.

#### Assessment of mt-HBT overall HCC sensitivity

All eligible and evaluable participants with a valid mt-HBT result and a confirmed HCC reference result will be included in the secondary analysis of mt-HBT overall HCC sensitivity. The analysis will include all confirmed HCC cases from both the ultrasound and CT/MRI screening arms and will be based on Visit 1 results. mt-HBT overall HCC sensitivity will be evaluated as the proportion of mt-HBT High results among participants with confirmed HCC status. A point estimate with 2-sided Wilson score 95% CI will be calculated for the mt-HBT overall HCC sensitivity.

#### Other prespecified analyses

mt-HBT positive and negative predictive values, positive and negative likelihood ratios, and area under the receiver operating characteristic curve will be calculated using the observed mt-HBT sensitivity and specificity, as well as HCC prevalence, where applicable. The impact of HCC prevalence on predictive values will also be assessed for varying prevalence values.

Point estimates with 95% confidence intervals will be calculated for mt-HBT HCC sensitivity and specificity, with stratification by tumor stage (eg, BCLC, Milan criteria), tumor size (<2 cm vs. 2–3 cm vs. 3–5 cm), and demographic and clinical characteristics (eg, body mass index, liver disease etiology, Child–Pugh score). The same performance metrics will be calculated and compared for AFP at various cutoffs, alone or combined with ultrasound.

mt-HBT sensitivity and specificity for indeterminate or suspicious observations (ie, LR-3, LR-4) will be calculated initially based on Visit 1 observations. After completion of Visit 2 and the full follow-up period, mt-HBT sensitivity and specificity will be recalculated for the observations that resolved to confirmed HCC or non-HCC status, using a range of allowable time intervals between blood collection and the resolved clinical status reference result. Similarly, the mt-HBT early-stage HCC sensitivity, overall HCC sensitivity, and HCC specificity will be recalculated after incorporating the resolved clinical status after the follow-up period, and compared with ultrasound, AFP, or ultrasound plus AFP.

Concordance and discordance between local and central radiologists, as well as between individual central radiologists, will be assessed for all CT/MRI images undergoing central radiology review with LI-RADS classification.

#### Missing data

All eligible and evaluable participants will be included in each analysis. Any patterns of missing data will be evaluated, and sensitivity analyses may be performed utilizing multiple imputation to assess the potential impact of missing data.

## DISCUSSION

This multicenter prospective validation study will provide novel data to inform the inclusion and positioning of the mt-HBT as a blood-based option among patients who meet established criteria for HCC screening. A major strength of the study is that all participants undergo standard-of-care screening imaging, blood collection for the mt-HBT, and contrast-enhanced multiphase cross-sectional reference imaging. In addition, the study employs a robust system for central radiology assessment in which every cross-sectional image is read by 2 independent fellowship-trained radiologists who are blinded to each other and to the local radiology reads. In case of discordance, differences are adjudicated by a third expert radiologist. Other strengths include the number and anticipated diversity of study sites and participants, robust sample size, and the direct comparative performance assessment of the mt-HBT against ultrasound for early-stage HCC sensitivity.

Within the context of the Early Detection Research Network (EDRN) 5-phase framework for biomarker development, phase 5 randomized controlled trials (RCTs) are generally considered the gold standard for establishing a reduction in cancer mortality to support widespread test adoption.^43^^,^^44^ A rationale for a phase 5 RCT is that demonstrating earlier detection alone may, in some cases, be insufficient to infer mortality reduction and other benefits. A recent study across cancer types reported that the correlation between late-stage cancer incidence and cancer-related mortality varies substantially by cancer type.^45^ Factors such as ineffective treatments, poor adherence, economic costs, morbidity associated with the test or diagnostic follow-up procedures, and overdiagnosis may contribute to a lack of overall benefit despite an observed cancer stage shift.^43^ However, HCC and the existing screening paradigm have specific characteristics that make a phase 5 RCT comparing screening with the absence of screening impractical. Notably, a significant HCC mortality benefit from screening of patients with chronic HBV infection has been demonstrated in an RCT.^46^ While no comparable trial exists for patients with cirrhosis, such a trial was attempted and found to be infeasible due to patient preferences for screening and the routine incorporation of screening recommendations into clinical care by providers, consistent with clinical practice guidelines.^47^ Contemporary trials are therefore more likely to evaluate the comparative performance of different screening modalities, and other trials such as those comparing ultrasound to abbreviated MRI or blood-based biomarker panels are ongoing.^48^^–^^51^

Several countries have implemented national screening programs from which real-world evidence suggests associations with early HCC detection and improved survival.^52^^–^^57^ Indications of likely benefits from screening have been demonstrated despite low adherence and sub-optimal early-stage sensitivity of ultrasound-based screening, both of which may potentially be improved upon by blood-based screening.^22^^,^^58^ Modeling of such improvements suggests substantial comparative benefits of mt-HBT screening on early-stage HCC detection and life years gained, with the potential for cost-effectiveness.^59^^,^^60^ While further study is needed to fully understand the extent of physical, psychosocial, and economic harms, analyses to date have not found the severity of harms to outweigh the perceived benefits of HCC screening.^22^^,^^61^^–^^65^ Specific aspects of existing guideline recommendations have also been developed to minimize harms associated with cross-sectional imaging and biopsy, as well as to minimize overdiagnosis.^61^ If satisfactory sensitivity and specificity characteristics of the mt-HBT can be demonstrated in ALTUS, it would support the likelihood that blood-based screening may improve early-stage HCC detection without substantially increasing harms. In addition, it may potentially help to reduce certain harms. For example, an understanding of biomarker profiles in participants with indeterminate imaging observations may enable risk stratification to optimize biopsy recommendations or additional screening.^61^ There are also indications that liquid biopsy tests such as the mt-HBT may be less likely than cross-sectional imaging to detect indolent cancers of low clinical significance, potentially reducing the risk of overdiagnosis.^61^^,^^66^^–^^69^ ALTUS will provide informative data to help address these and other important questions about blood-based screening.

The ALTUS study design will provide high-quality evidence on the performance and potential value of the mt-HBT. Of note, similar prospective cross-sectional screening studies have been successfully conducted for regulatory and reimbursement assessment of novel biomarker tests designed to detect other cancers.^70^^–^^73^ All participants in such studies generally undergo prospective sample collection along with diagnostic procedures to establish reference results for the test’s operating characteristics (ie, sensitivity and specificity). ALTUS represents a version of this study design and has both advantages and limitations relative to other approaches, such as retrospective repository studies or prospective clinical utility studies that limit diagnostic procedures to patients with positive test results. A major strength of ALTUS is the ability to efficiently and rigorously assess sensitivity and specificity in a large, prospective sample from the intended use population. In addition, tumor characteristics near the time of sample collection can be evaluated for both test-positive and test-negative cases, enabling subgroup performance analyses by tumor size and other metrics. In contrast, retrospective repository studies must accumulate samples and wait for clinical diagnoses to establish cases and controls for analysis, and tumor characteristics may not be known at the time a biomarker test becomes positive. Similarly, performance estimates in repository studies and clinical utility studies may be less accurate without all participants receiving diagnostic procedures to establish true negative and false negative test results. However, it is acknowledged that ALTUS does not assess how mt-HBT results may impact real-world longitudinal clinical management of patients, as results are not returned to providers in the study. In addition, it is possible that the application of diagnostic cross-sectional imaging to all participants may result in the detection of a disproportionate number of very-early HCCs (<2 cm), which are less likely to be detected by ultrasound or blood-based tests. In this scenario, the clinical significance of the apparent ultrasound and mt-HBT HCC sensitivity may be more challenging to interpret relative to sensitivity for larger HCCs that are still early-stage but at greater risk of consequential progression before the next screening interval. This emphasizes the importance of understanding performance by tumor size and highlights the potentially large impact of differences in screening adherence.

There is an urgent need to address the substantial public health burden imposed by HCC. Imaging-based screening modalities can be onerous and associated with low patient adherence, whereas a blood-based test may facilitate the incorporation of HCC screening in the current workflow of patients with cirrhosis, who routinely undergo blood tests to assess liver function. The available evidence for the utility of HCC screening, together with the potential for improvement over imaging-based screening paradigms, underscores the relevance and importance of the ALTUS study. Demonstration of favorable mt-HBT performance relative to ultrasound using a robust prospective study design may advance support for blood-based screening as a viable approach to improve outcomes for patients at elevated risk for HCC.
